# A sensitive probe for amyloid fibril detection with strong fluorescence and early response†

**DOI:** 10.1039/c8ra00751a

**Published:** 2018-04-27

**Authors:** Xiaolin Zheng, Zhenzhen Xu, Haiyang Li, Hongbing Fu

**Affiliations:** Beijing Key Laboratory for Optical Materials and Photonic Devices, Department of Chemistry, Capital Normal University Beijing 100048 People's Republic of China xuzhenzhen@cnu.edu.cn hbfu@cnu.edu.cn; College of Life Sciences, Capital Normal University Beijing 100048 People's Republic of China; Tianjin Key Laboratory of Molecular Optoelectronic Sciences, Department of Chemistry, School of Sciences, Tianjin University, Collaborative Innovation Center of Chemical Science and Engineering Tianjin 300072 People's Republic of China

## Abstract

We synthesized a new probe, 4-[2-(2-naphthyl)-(*E*)-ethenyl]-benzyl(triphenyl)phosphonium bromide (NEB), to detect the formation of amyloid fibrils of bovine insulin. The fluorescence intensity of NEB in the presence of insulin fibrils was 30 times higher than that before fibrillation, with the fluorescence quantum yield increased from 2.5% to 78%. In comparison with the commercially available probe, thioflavin T (ThT), NEB exhibits a 10 times stronger fluorescence and a shorter identification lag phase for detecting insulin fibrillation, indicating a higher sensitivity in detection of insulin oligomers and fibrils.

## Abstract 2

We synthesized a new probe, 4-[2-(2-naphthyl)-(*E*)-ethenyl]-benzyl(triphenyl)phosphonium bromide (NEB), to detect the formation of amyloid fibrils of bovine insulin.

## Graphical abstract

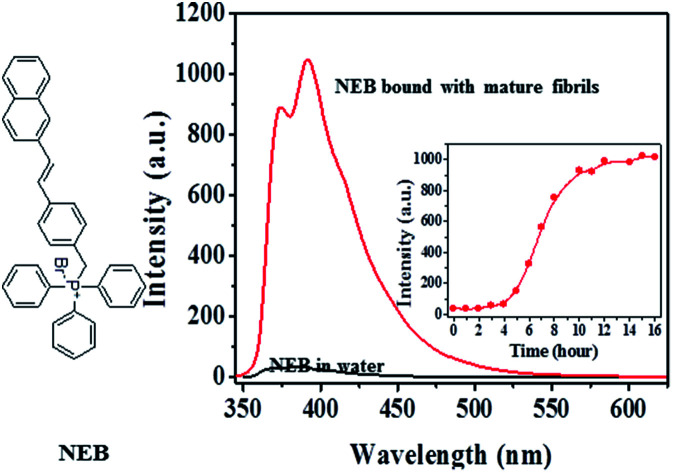

## Introduction

**Fig. 1:**
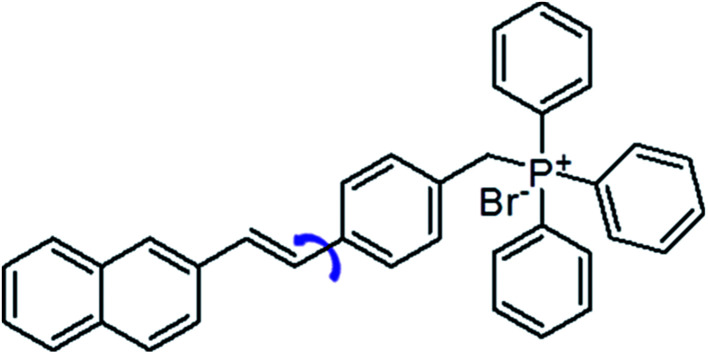
Chemical structure of NEB.

Amyloid fibrils are a denatured fibrous form of protein aggregates, and have been extensively investigated because their deposition causes several serious diseases, such as Alzheimer's and Parkinson's diseases, type II diabetes and prion diseases.^1^ Note that the fibrils formation is not only limited to the protein related to the neurodegenerative diseases. It has been widely accepted that the fibril formation is an intrinsic property of the polypeptide backbones.^2^ Potentially, any protein could form amyloid fibrils under specific conditions.^3^ The fibril structure appears generic linear filaments with a length of several micrometres and a width about 10 nm. Fibril X-ray diffraction studies have revealed an antiparallel β-sheets oriented perpendicular to the long axis of the fibril.^4^ As these fibrils and/or amyloid fibril intermediates (oligomers) can kill cells or prevent them from functioning properly, detecting these fibrils and understanding the mechanism of the fibril formation are important for the drug development against these neurodegenerative diseases.^5^ Recently, different characterization techniques of amyloid fibrils have been developed, such as absorption, circular dichroism spectra, mass spectrometry and atomic force microscopy as well as fluorescence.^6^ Among them, fluorescence is the most popular one, as it allows a direct observation of the whole fibrillation process.^6^ In the past decades, a great variety of fluorescent probes have been developed to monitor the fibrillation process, including small organic molecules, gold nanoparticles, fluorescent proteins and conjugated polymers.^2,7^ In particular, thioflavin T (ThT) is a well-known probe for amyloid fibrils detection with several advantages. (i) ThT molecules are water-soluble and are extensively used both *in vitro* and *in vivo*.^8^ (ii) ThT interacts only with the mature fibrils and does not interfere with the amyloid fibrillation process.^9^ (iii) When bound to the amyloid fibrils, the ThT fluorescence increases significantly.^10^ Nonetheless, detecting the oligomeric of the proteins which is the main contributor of the neurotoxicity is still a great challenge for the ThT. On the other hand, the low fluorescence intensity is another major drawback for the ThT probe. In these regard, great attention have been paid in searching for efficient dyes with better performance. According to the research efforts of scientists, the structure of molecular rotors are particularly interesting for use as amyloid binding agents due to their distinct spectral changes depending on environmental factors and an increase in quantum efficiency upon being geometrically confined.^11^ For example, Catherine C. Kitts *et al.* demonstrated that Michler's hydrol blue is an excellent amyloid fibril probe, which exhibits a characteristic red-shift in its excitation spectrum and an increase in the emission quantum yield upon binding to the amyloid fibrils.^12^ Ben Zhong Tang *et al.* have reported a dye TPE–TPP as a probe to monitor the α-synuclein (α-Syn) fibrillation process which shows a better performance over the ThT on the emission intensity and the sensitivity towards the oligomeric form of the α-Syn.^13^ 4-[2-(2-Naphthyl)-(*E*)-ethenyl]-benzyl(triphenyl)phosphonium bromide (NEB) is a typical molecular rotor which has been known as the synthetic intermediates of the carbohelicenes (Fig. 1A).^14^

In this work, we will report the optical characterization of NEB to monitor amyloid fibril formation. We will demonstrate that NEB is indeed an excellent amyloid fibril probe. The NEB exhibits the weak fluorescence in the solution of the native protein but exhibits an increase in quantum efficiency when binding to the mature fibrils of bovine insulin. Compared to ThT, NEB exhibits a 10 times stronger fluorescence intensity upon binding to the insulin fibrils which arise from the increased the *k*_r_ and decreased *k*_nr_. The NEB is utilized to monitor the insulin fibrillation process and demonstrated a good amyloid fibril probe with a shorter identification lag phase for detecting insulin fibrillation.

## Experimental section

### Materials and instruments

NEB was synthesized and confirmed by mass spectrometry (GCT-MS Micromass, UK) and ^1^H NMR. Thioflavin T (ThT) and insulin from bovine pancreas were purchased from Sigma-Aldrich with no further purification.

Insulin amyloid fibrils were prepared according to the protocols described elsewhere.^12^ In the study of using NEB and ThT as an ex situ probe, an aliquot of the insulin solution taken out from the incubation mixture at a defined time was diluted with Tris–HCl buffer, followed by the addition of the probe. In the study of using NEB and ThT as an *in situ* probe, they were added to insulin solution prior to incubate at 60 °C with constant agitation at 600 rpm. The final concentrations of insulin, NEB and ThT were 5 μM, 2 μM and 2 μM, respectively.

The steady-state absorption spectra were measured on a Shimidazu UV-3600 UV-VIS-NIR spectrophotometer. The stationary fluorescence spectra were performed on a Horiba FluoroMax-4-NIR spectrophotometer equipped with an integrating sphere. The relative fluorescence quantum yields of solutions and colloidal suspensions were measured by using 9,10-diphenylanthracene as a reference (*Φ* = 0.95).

## Results and discussion

**Fig. 2:**
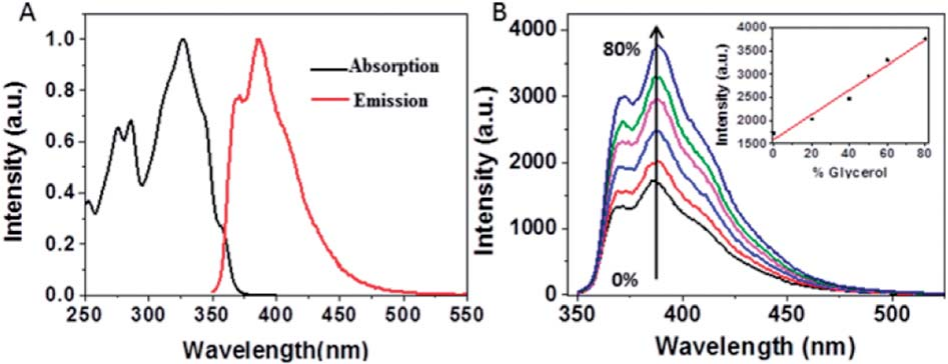
(A) Normalized absorption (red) and emission (black) spectra of NEB alone in ethanol solution. (B) Emission spectra of NEB in different ratios of ethanol: glycerol. 100 : 0 (black), 80 : 20 (red), 60 : 40 (blue), 50 : 50 (pink) 40 : 60 (green), 20 : 80 (dark blue), inset: plot of the emission intensity for each solution *versus* percentage glycerol present.

Fig. 2A presents the steady-state absorption (black line) and fluorescence (red line) spectra of NEB in the dilute ethanol solution. The lowest S_0_ → S_1_ transition of NEB monomers in the dilute ethanol solution is a broad and structured band with a molar extinction coefficient of 33 263 M^−1^ cm^−1^ at 325 nm. The monomer fluorescence spectrum exhibits a vibronic progression, with the maximum at 384 nm. To verifying that NEB belongs to a class of molecular rotors,^15^ we dissolved NEB in a mixture of ethanol and glycerol. The concentration of glycerol was varied from 0% to 80% to increase the viscosity. It can be seen from Fig. 2B that the emission intensity of NEB in the ethanol/glycerol solution show a linear increase with increasing the viscosity. The low florescence intensity of NEB in 100% ethanol can be ascribed to the torsional relaxation around C–C bond in the excited state. However, upon increasing the viscosity, the intramolecular rotation is greatly restricted. As a result, the NEB molecules become highly emissive in solvent of high viscosity.

Upon excitation in ethanol, NEB goes from a locally excited state to a non-fluorescent TICT state upon internal rotation around C–C bond. This internal rotation which nonradiatively annihilates the excited state is much faster than the fluorescence decay, making NEB free in solution exhibit weak fluorescence intensity. However, upon increasing the viscosity of the solution, the intramolecular rotation is greatly restricted. The restriction of intramolecular rotation blocks relax into the TICT state and make the excited state remain in the LE state which gives off its energy in the form of fluorescence. As a result, the NEB molecules become highly emissive in solvent with high viscosity. Notably, NEB is water-soluble with a saturation concentration of 1 × 10^−5^ M. These unique properties make NEB attractive as a probe for amyloid fibril structure determination *in vitro* and *in vivo*.

**Fig. 3:**
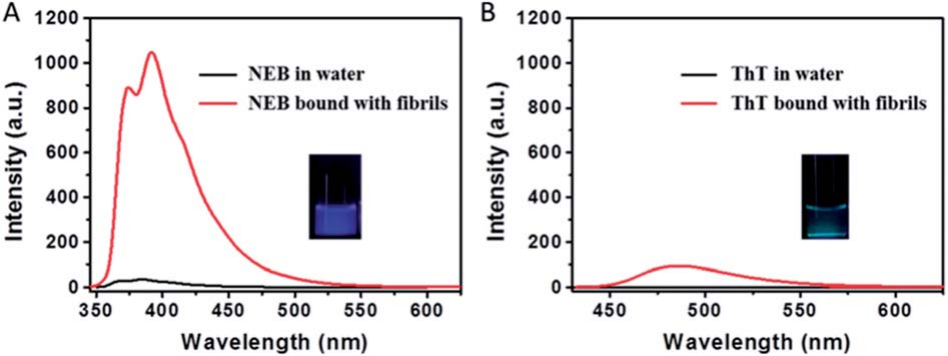
Emission spectra of (A) NEB (2 μM) and (B) ThT (2 μM) dissolved in water (black) and mature fibrils solution (red), respectively. *λ*_ex_ = 325 nm (for NEB), 410 nm (for ThT). Photographs of mixtures of NEB and ThT with fibrillar forms of insulin taken under UV illumination.

Insulin fibrils were prepared by dissolving the protein in water solution (0.01 M HCl, 5 mg mL^−1^ for insulin) and incubated in an orbital thermomixer with constant agitation at 600 rpm at 60 °C for 24 h. We used ThT as probe to detect the mature insulin amyloid fibrils. After 24 h incubation, the emission signal from ThT at 487 nm greatly increases, indicating the formation of mature insulin amyloid fibrils (Fig. 3).

The transmission electron microscopy (TEM) in Fig. S1† shows that mature fibrils have a length of several micrometres with an average width of 7–10 nm. When the mature insulin fibrils were introduced into the solution with the NEB, distinct spectral changes were observed compared to that of NEB in water: (i) The absorption spectrum has a 9 nm red-shift from 324 nm to the 333 nm (Fig. S2†). (ii) The fluorescence spectrum also exhibits a 7 nm red-shift from 384 nm to the 391 nm (Fig. S2†). (iii) When the two un-normalized spectra were compared in Fig. 3A, enhanced fluorescence emission signal of NEB is observed in the presence of mature insulin fibrils. The fluorescence intensity for the NEB in the presence of insulin fibrils was 30 times higher than that of NEB with insulin before fibrillation with a fluorescence quantum yield increased from the 2.5% to the 78%. These distinct spectral changes indicate that NEB is capable of binding to the insulin fibrils and detecting the mature amyloid fibrils. To make sure that NEB is binding specifically to insulin fibrils and not to native protein, we compared the absorption and fluorescence spectra of NEB containing insulin before fibrillation with that of NEB in water solution and no difference is found (Fig. S3†). Thus, we can predict that NEB binds specifically to the β-sheet structure of the amyloid fibrils in the same way as ThT. We have measured the absorption and emission spectra of NEB in different solvents. It can be seen that the maximum absorption peak shifts from 324 nm (water) to 331 nm (toluene), showing a red-shift of 5 nm. Correspondingly, the fluorescence emission maximum shifts from 384 to 389 nm with reducing polarity of the solvent (Fig. S4†). So the red-shift of the absorption and fluorescence spectra of NEB from in water to bound with fibrils can be ascribed to the reduced polarity at the NEB binding site in the insulin amyloid fibrils.

The dramatic increase of fluorescence of NEB when bound to insulin fibrils is similar to that of when NEB in high viscosity solvent. The increase in emission intensity of NEB upon binding to the fibrils also arises from the restriction of the NEB's internal rotation around the C–C bond. Excitingly, NEB displays fluorescence intensity more than 11 times stronger than ThT under the same conditions. The stronger fluorescence intensity is also observed clearly in the photographs of insulin amyloid fibrils stained by NEB and ThT taken under a UV lamp (365 nm) in Fig. 3.

**Fig. 4:**
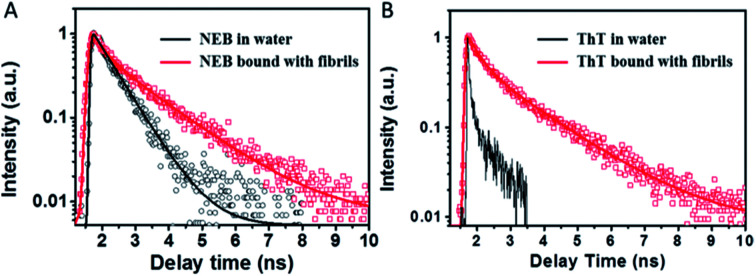
Fluorescence decay profiles of NEB (A) and ThT (B) in water solution (black) and mature fibrils solution (red), respectively.

In order to understand the fluorescence enhancement mechanism, we measured the lifetime data of NEB and ThT in the water alone and in solution with mature insulin fibrils. ThT bound to insulin fibril at 452 nm exhibits bi-exponential fluorescence decay with an average excited-state lifetime 1.02 ns containing a lifetime of 0.44 ns (A1 = 0.53) and a slower component 1.68 ns (A2 = 0.47). The ThT in the insulin before fibrillation decays monoexponentially with a faster lifetime < 10 ps (Fig. 4). These are all consistent with earlier reports.^16^ However, the lifetime could not be calculated accurately because of the limitation by the resolution of our apparatus. Thus, the three order of magnitude slower lifetime of ThT bound to insulin fibril compared to the ThT in water leads to its dramatic increase of the fluoresce intensity.^8^

The NEB in water follows single exponential decay with *τ* = 0.50 ns. After fibrillation, the time resolved fluorescence decays of the NEB bound to insulin fibrils was represented by a double-exponential fit with a lifetime of 0.42 ns (A1 = 0.43) and a slower component 1.86 ns (A2 = 0.57). The average excited-state lifetime is 1.24 ns. Then we calculated the radiative decay rates (*k*_r_) and nonradiactive decay rates (*k*_nr_) based on equations of *k*_r_ = *Φ*/*τ* and *Φ* = *k*_r_/(*k*_r_ + *k*_nr_). It can be seen from Table 1 that the value of *k*_r_ increases almost one order of magnitude from *k*_r,water_ = 0.05 ns^−1^ to *k*_r,fibrils_ = 0.62 ns^−1^; meanwhile, the value of *k*_nr_ decreases one order of magnitude from *k*_nr,water_ = 1.95 ns^−1^ to *k*_nr,fibrils_ = 0.17 ns^−1^. Therefore, the greatly enhanced fluorescence can be ascribed to the increase of the *k*_r_ as well as the decrease of the *k*_nr_ when bound to the mature insulin fibrils.

**Table 1:** Photophysical data for NEB in water and bound to fibrils

	*λ*_abs_ a (nm)	*ε* b (M^−1^cm^−1^)	*λ*_em_ c (nm)	*Φ* d (%)	*τ* e (ns)	*k*_r_ f (ns^−1^)	*k*_nr_ g (ns^−1^)
NEB in water	327	33263	384	2.5	0.50	0.05	1.95
NEB bound to fibrils	333	—	392	78	1.24	0.62	0.17

a The maximum absorption wavelength.

b The molar extinction coefficient.

c The maximum emission wavelength.

d Fluorescence quantum yield.

e Lifetime.

f The radiative decay rates.

g The non-radiative rates.

The dissociation constants (*K*_d_) of NEB and ThT were derived by using a fixed concentration of the mature insulin fibrils with varying dye concentrations and fitted using a one site binding equation. The resulting *K*_d_ for NEB and ThT are 3.36 μM and 6.68 μM, respectively (Fig. S5†). According to reports, ThT binds into channels formed by side chains of the residues of β-strands on the surface of the β-sheet of the fibril, and binding is predominantly along the fibril axis.^17^

**Fig. 5:**
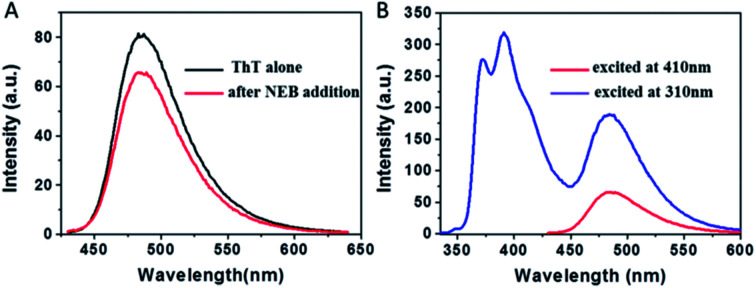
(A) The emission spectra of ThT bound with insulin amyloid fibrils (black) before and (red) after the addition of NEB, *λ*_ex_ = 410 nm. (B) The emission spectra of ThT bound with insulin amyloid fibrils after addition of NEB at different excitation wavelength, *λ*_ex_ = 310 nm (blue), *λ*_ex_ = 410 nm (red).

To examine whether NEB and ThT have the same mode of binding to amyloid fibril, a new experiment was performed. We added ThT into the amyloid fibrils' solution, and then added the NEB into the solution after ThT binding to the fibrils. We defined the sample as NEB @ ThT, the concentration of NEB and ThT in NEB @ ThT are all 2 μM. The fluorescence emission signal from ThT is significantly reduced in the presence of NEB (Fig. 5A), indicating that NEB may compete for the same binding sites with ThT. Considering that this excited wavelength of NEB could not excite the ThT, and the NEB themselves display bluish emissive upon UV excitation (Fig. 3A), with a broad spectrum covering 350–500 nm that overlaps absorption spectrum of ThT (Fig. S6†), energy transfer may takes place from excited NEB to ThT. Under the excitation wavelength 310 nm of the NEB, we collected the emission signal of 487 nm typical of ThT and the emission signal of 391 nm typical of NEB at the same time. Moreover, the emission signal of the ThT excited at 310 nm exhibits a higher intensity than that at the excitation wavelength 410 nm. This result suggest that efficient energy transfer takes place from excited NEB to ThT in NEB @ ThT bound with mature fibrils.

The absorption spectrum of the sample NEB @ ThT exhibits the absorption peaks with a strong peak at 325 nm ascribed to the NEB and a relatively weaker peak at 410 from the ThT (Fig. S7†). Fluorescence excitation spectrum monitored at the ThT emission (520 nm) agrees well with the absorption spectrum, revealing energy levels including not only the NEB but also ThT. This is another piece of clear evidence for the energy transfer from the donor (NEB) to the acceptor (ThT). Furthermore, the process of energy transfer affected the fluorescence decay curve of the donor NEB. The fluorescence of NEB bound to insulin fibrils alone decays double-exponential fit with a lifetime of 0.42 ns (A1 = 0.43) and a slower component 1.86 ns (A2 = 0.57) (Fig. 4A and S7B,† red line). However, the fluorescence decay of NEB in the NEB @ ThT bound to mature insulin fibrils becomes faster with an average lifetime of 0.51 ns, which would remain unchanged if energy transfer occurred *via* radiative mechanism, indicating that binding to the fibrils make sure the proximity of the NEB and ThT. This is consistent with the spacing of β-sheets which formed the binding sites for ThT and NEB is about 0.65–0.7 nm. It is reported that the main interactions between ThT and amyloid fibrils are hydrophobic effect and π-stacking which does not have an effect on the fibrillation kinetics. Considering that NEB has the similar binding sites with the ThT, we can make a prediction that NEB would not affect the fibrillation processes.

**Fig. 6:**
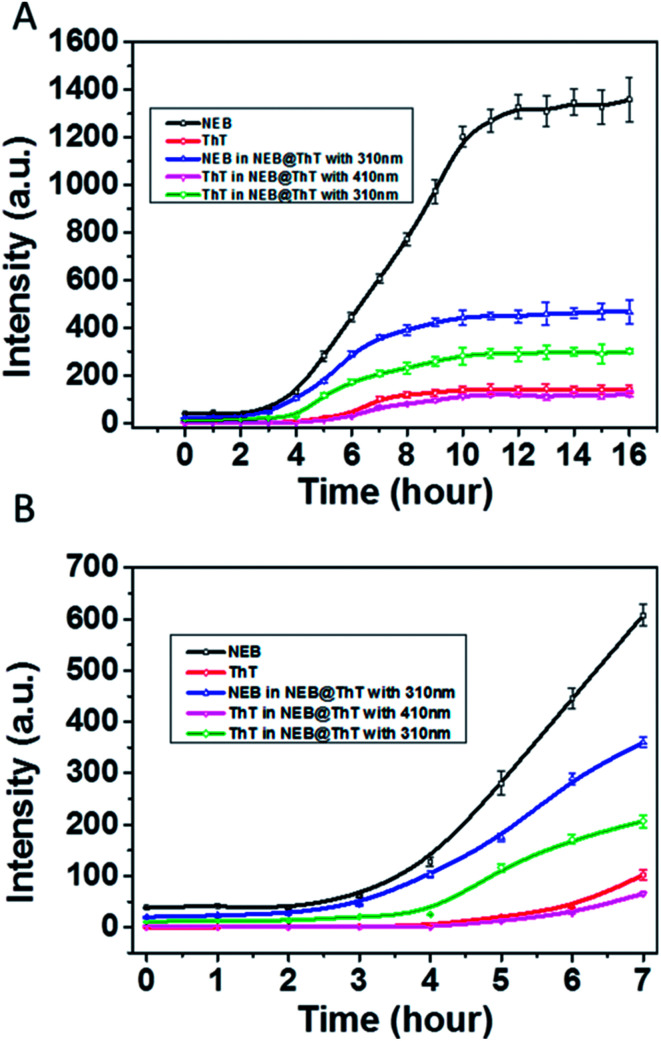
(A) Emission intensity of NEB (black, 2 μM) and ThT (red, 2 μM) monitoring insulin (5 μM) fibrillation, *λ*_ex_ = 325 nm (for NEB), 410 nm (for ThT). NEB and ThT monitoring insulin fibrillation at the same time, the emission of intensity of NEB (blue, *λ*_ex_ = 310 nm), ThT (green, *λ*_ex_ = 310 nm) and ThT (pink, *λ*_ex_ = 410 nm). (B) The enlarged part of insulin fibrillation from *t* = 0 to 7 h.

We then used NEB and ThT to monitor the amyloid fibrillation processes, respectively (Fig. 6). The maximum emission intensity of the NEB and ThT were utilized. Through following the emission intensity of the NEB at 391 nm, the amyloid fibrillation can clearly be monitored by the fluorescence enhancement. The fluorescence intensity of the samples incubated for 12 to 16 h increased about 30 times compared to the sample before incubation, which is in good agreement with the results obtained from the experiment of adding the NEB into the mature fibrils directly in Fig. 3A. Moreover, the lifetime of the NEB during the incubation process was performed. After incubating for 6 h, the time resolved fluorescence decays of the NEB bound to insulin fibrils was also represented by a double-exponential fit with a lifetime of 0.41 ns (A1 = 0.78) and a slower component 1.82 ns (A2 = 0.22). The average excited-state lifetime is 0.72 ns. (Fig. S8†) Comparing to the NEB in water alone, the value of *k*_r_ was increased to 0.42 ns^−1^ with the value of *k*_nr_ was decreased to 1.0 ns^−1^. These results verified that the fluorescence enhancement by the restriction of the NEB's internal rotation around the C–C bond when bound to the insulin fibrils. An initial lag phase, an exponential growth phase, and a final plateau phase are present in the Fig. 6, which is in good agreement with the results obtained from the similar experiment using ThT. NEB exhibits a stronger emissive during the whole process of the fibrillation compared with the fluorescence from the ThT (Fig. 6, red line). Closer observation of the kinetics of the insulin fibril formation reveals that there is an earlier response to the lag phase of insulin fibrillation by NEB than ThT. At *t* = 2 h after incubation, all the emission of probes only show background signals. Fluorescence enhancements were observed at *t* = 2 h with NEB and at *t* = 4 h with ThT. As is reported, the ThT only interacts with the mature fibrils. The earlier response of the NEB in Fig. 6 indicates that NEB is capable of detecting the formation of an intermediate which is significantly different from the monomeric and fibrillar forms of the insulin.

Considering that energy transfer could take place from NEB to ThT, another experiment was performed. ThT and NEB were added into the solution successively with different incubation times. The concentrations of NEB and ThT in the NEB @ ThT system are all 2 μM. The emission intensity of NEB in NEB @ ThT with excitation wavelength 310 nm (391 nm, blue line in Fig. 6A), ThT with excitation wavelength 310 nm (487 nm, green line in Fig. 6A) and 410 nm (487 nm, violet line in Fig. 6A) were utilized respectively with different time points during the formation of amyloid fibrils of insulin. Compared with NEB alone (black line in Fig. 6), every phase of NEB in NEB @ ThT is in good agreement with the result of NEB alone. However, the fluorescence emission intensity of NEB is reduced in the presence of ThT, which is ascribed to the energy transfer from NEB to ThT. For the ThT in NEB @ ThT (Fig. 6, violet line), it also show the similar “S” curve with a decreased fluorescence intensity, which agrees well with the result of the competition experiment in Fig. 5B. The fluorescence emission signal of ThT (Fig. 6, green line) excited at 310 nm also produced a “S” curve with three different phase. Closer observation of the emission spectra reveals that the fluorescence enhancement was observed at *t* = 4 h. The 2 h lag time compared to NEB alone is ascribed to that the amount of ThT bound with insulin is not enough for energy transfer at this moment, which in accordance with that NEB might detect the oligomeric of the proteins which is still a challenge for ThT. Recently, it is reported that the oligomer form of protein, rather than the fibrillar form, is the main contributor of neurotoxicity.^18^ The earlier response with the stronger fluorescence intensity mean the NEB will be a potential and promising candidate fluorescent probe for fibrillation detection and early stage detection of the amyloid diseases.

## Conclusions

In conclusion, we have demonstrated a new blue-emissive, high fluorescence intensity, earlier response probe NEB for monitoring amyloid fibrillation process. The NEB exhibits the weak fluorescence in the solution of the native protein but exhibits great increase in quantum efficiency when bound to the mature fibrils of bovine. A thorough photophysical study has shown that the enhanced fluorescence arise from the increased the *k*_r_ and decreased *k*_nr_. Compared to ThT, NEB exhibits a 10 times stronger fluorescence and a shorter identification lag phase for detecting insulin fibrillation, indicating a higher sensitivity in detection of insulin oligomers and fibrils. Considering the short emission wavelength of the NEB, the tailoring of the molecular structures for the red-shift of the emission spectrum was needed. This work is under way in our laboratories and will be published elsewhere.

## Conflicts of interest

There are no conflicts to declare.

## Supplementary Material

RA-008-C8RA00751A-s001
