# Supplementary material for: A sensitive probe for amyloid fibril detection with strong fluorescence and early response
Source: RSC Adv. 2018 Apr 27;8(29):15870–5. doi: 10.1039/c8ra00751a (PMC9080103; doi:10.1039/c8ra00751a)
Supplement: RA-008-C8RA00751A-s001 [file RA-008-C8RA00751A-s001.pdf]

Electronic Supplementary Material (ESI) for RSC Advances.
This journal is © The Royal Society of Chemistry 2018

Supporting Information

A sensitive probe for amyloid fibril detection with strong fluorescence and early Response

Xiaolin Zheng,[ab] Zhenzhen Xu,*[a] Haiyang Li[a] and Hongbing Fu*[ac]

a. *Beijing Key Laboratory for Optical Materials and Photonic Devices, Department of Chemistry, Capital Normal University,Beijing 100048, People's Republic of Chinab.*
b. *College of life sciences, Capital Normal University,Beijing 100048, People's Republic of China.*
c. *Tianjin Key Laboratory of Molecular Optoelectronic Sciences, Department of Chemistry, School of Sciences, Tianjin University, Collaborative Innovation Center of Chemical Science and Engineering, Tianjin 300072, People's Republic of China*

E-mail : hbfu@cnu.edu.cn; xuzhenzhen@cnu.edu.cn

Experimental

Materials

All reagents for organic synthesis were purchased from Alfa Aesar and used as received without further purification. Other organic solvents (HPLC grade) were purchased from Beijing Chemical Agent Ltd., China. Ultrapure water with a resistivity of 18.2 MΩ cm-1, produced using a Milli-Q apparatus (Millipore), was used in all experiments.

Synthesis and characterization of NEB

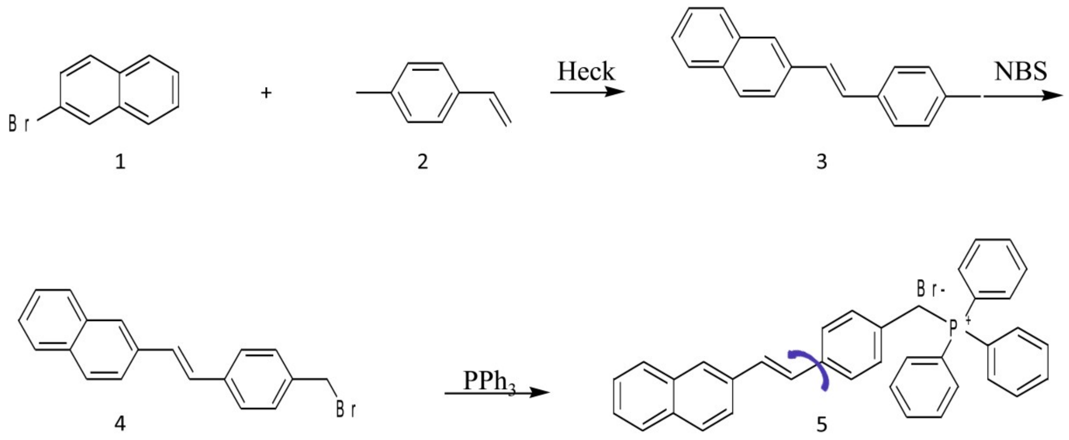

Synthetic routes of the compound of **NEB.**

A mixture of 2-Bromonaphthalene (2 g, 9.7 mmol), 4-Methylstyrene (1.14 g, 9.7 mmol), Pd(OAc)2 (110 mg, 0.49mmol), triphenyl phosphate (1.29, 3.9 mmol), tri-n-butylamine (11 g, 58.8 mmol), Dimethyformamide (20 mL) was introduced into a pressure tube, under argon atmosphere. The reaction mixture was heated for 24 h at 130 ℃ and then poured into water. The phase was extracted with CH2Cl2. The pooled organic phases were washed with water, dried over anhydrous MgSO4, filtered, and evaporated. The product was separated by flash chromatography on silica gel by means of CH2Cl2/petroleum ether (1:1). Finally molecular 3 was obtained.

The molecular 3 was characterized by 1H NMR, ^{13}C spectroscopy.

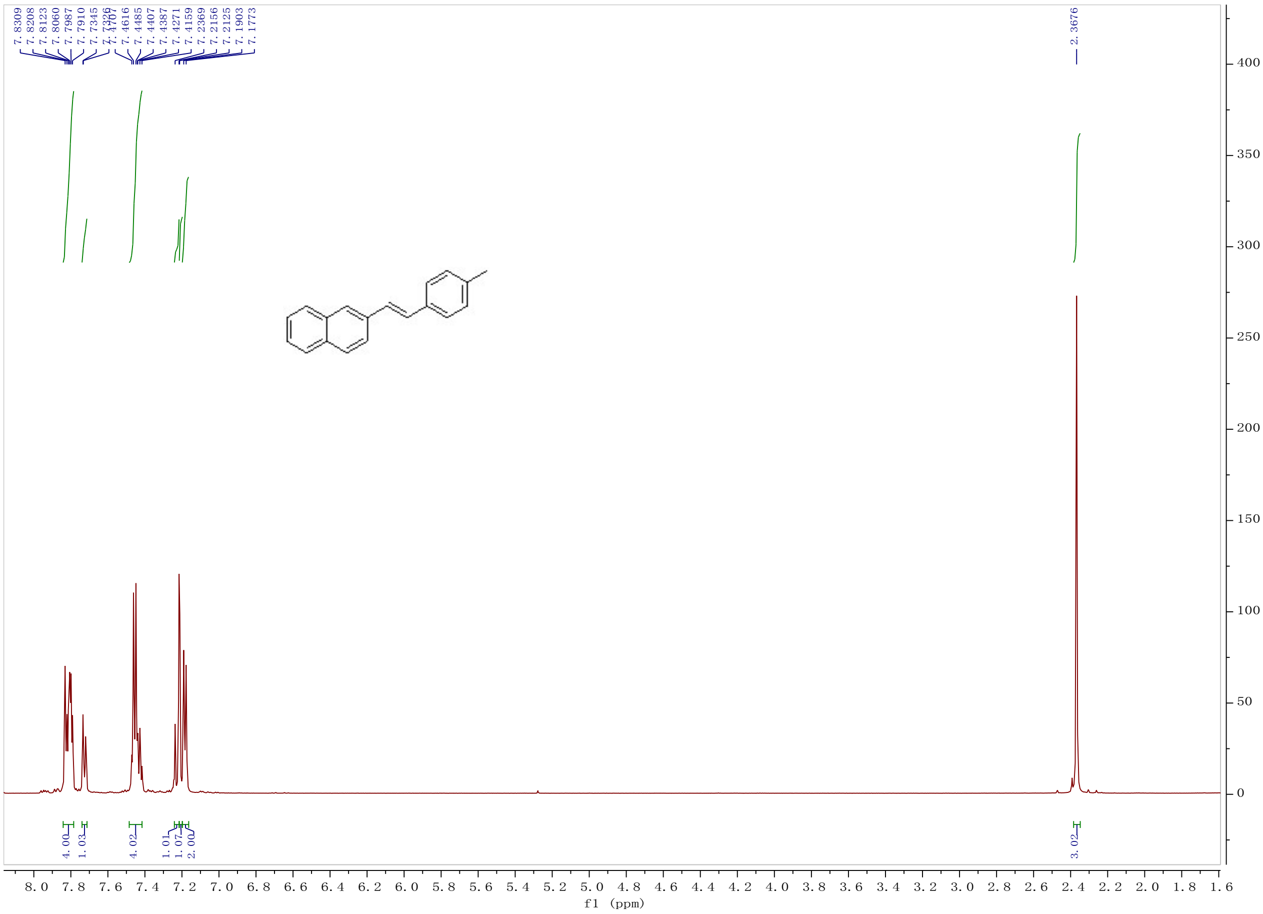

^{1}H NMR (600 MHz, $CDCl_3$) δ: 7.83–7.79 (m, 4H), 7.73 (dd, J = 8.4, 1.2Hz, 1H), 7.47–7.41 (m, 4H), 7.23 (d, J = 15.0 Hz, 1H), 7.20 (d, J = 15.0 Hz, 1H), 7.18 (d, J = 8.4 Hz, 2H), 2.39 (s, 3H).

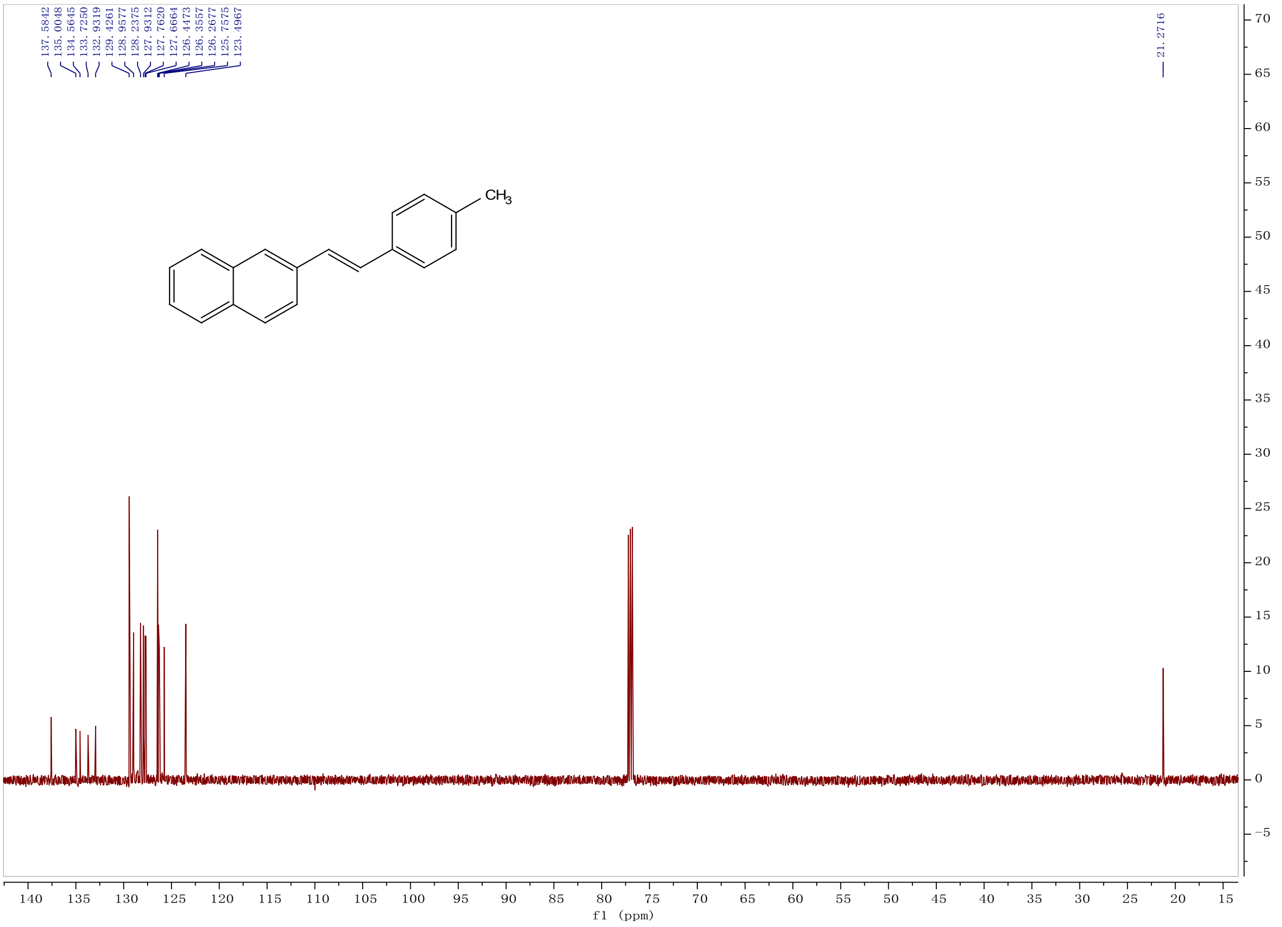

^{13}C NMR (151 MHz, $CDCl_3$) δ: 137.58, 135.00, 134.56, 133.73, 132.93, 129.43, 128.96, 128.24, 127.93, 127.76, 127.67, 126.45, 126.36, 126.27, 125.76, 123.50, 21.27.

Then N-Bromosuccinimide (854 mg, 2.4 mmol) and dibenzoyl peroxide (40 mg) was added to a refluxing solution of molecular (1g, 4.1 mmol) in carbon tetrachloride (100 mL). The solution was refluxed for 72 h. After cooling, the product was used with further purification by flash chromatography on silica gel, we get the molecular 4. To a solution of the molecular 4 (200 mg, 0.6 mmol) in toluene (20 mL) was added triphenylphosphine (160 mg, 0.61 mmol) and the solution refluxed for 12 h to produce a white suspension. After filtration at room temperature, the beige solid was washed with petroleum ether and dried under vacuum to get the pure molecular 5.

The molecular 5 was characterized by MS (mass spectrometry), 1H NMR spectroscopy

Mp = 275–277 °C.

MS (ESI) 505.24

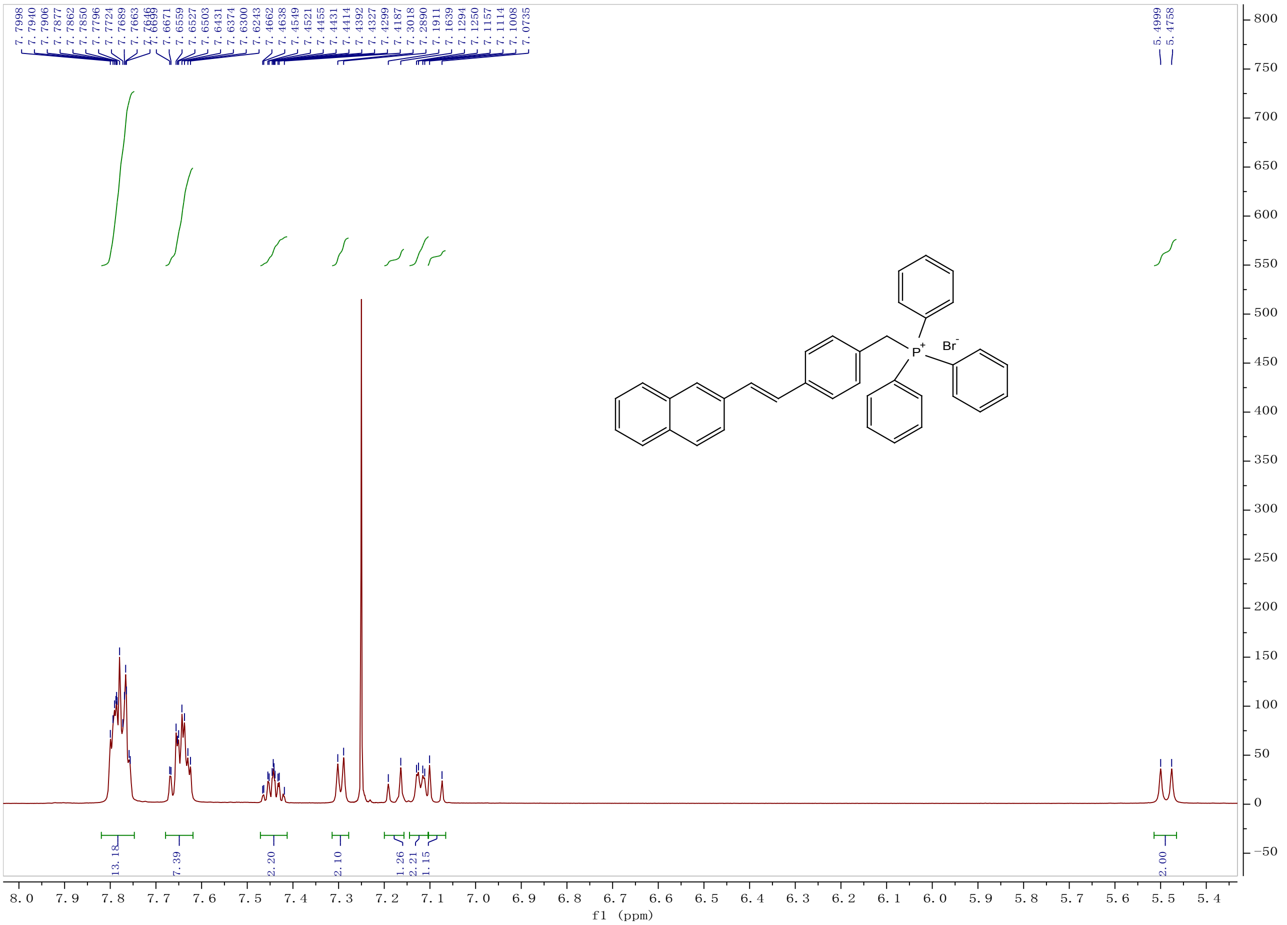

^{1}H NMR (600 MHz, $CDCl_3$) *δ*: 7.75-7.80 (m, 13H), 7.62-7.67 (m, 7H), 7.42-7.47 (m, 2H), 7.30 (d, *J* = 7.7 Hz , 2H), 7.11-7.13 (m, 2H), 7.18 (d, *J* = 16.3 Hz , 1H), 7.09 (d, *J* = 16.3 Hz , 1H), 5.48 (d, *J* = 14.5 Hz , 2H).

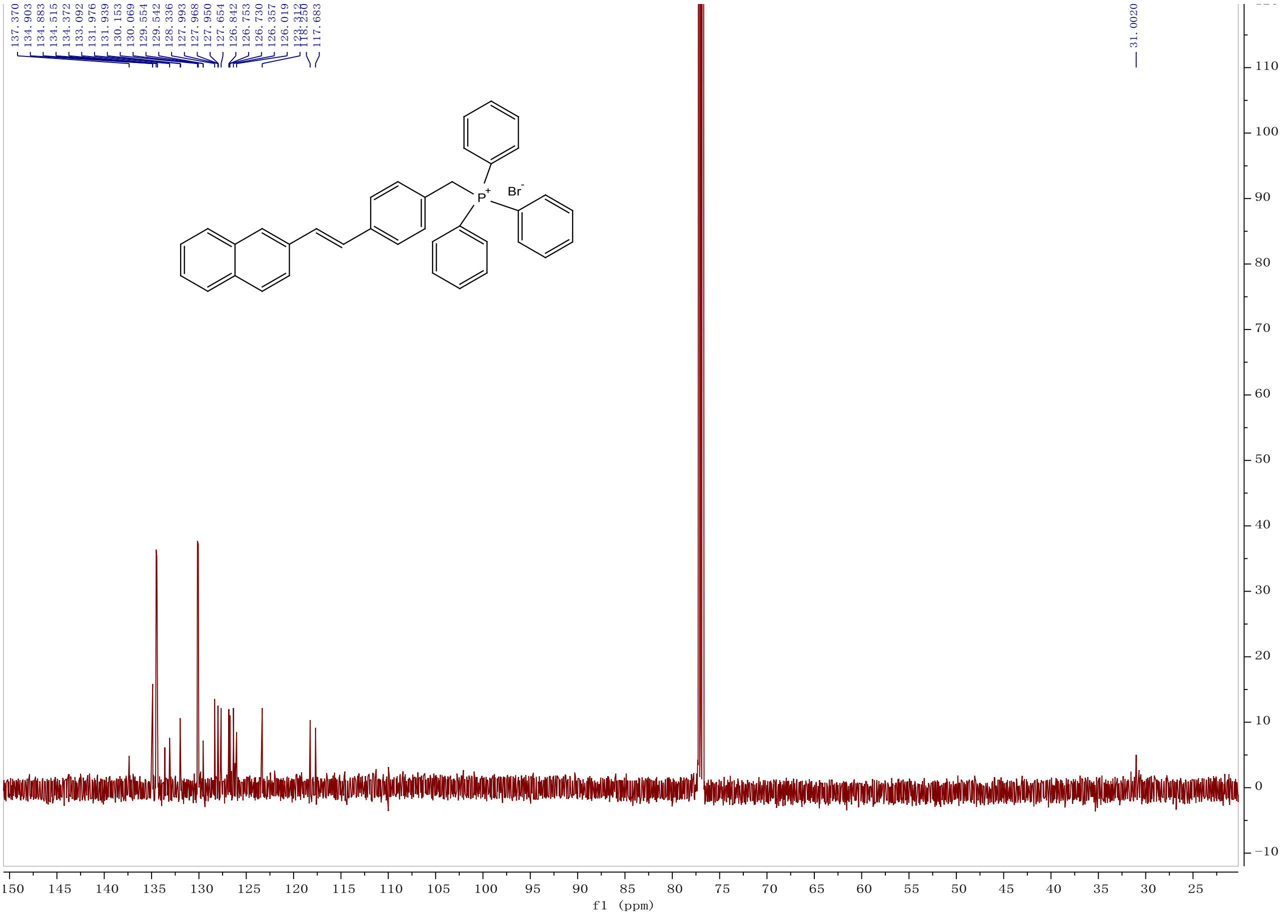

^{13}**C NMR** (151 MHz, $CDCl_3$) δ: 137.37, 137.34, 134.90, 134.88, 134.52, 134.48, 134.37, 133.62, 133.09, 131.98, 131.94, 130.15, 130.07, 129.55, 129.54, 128.34, 127.99, 127.97, 127.95, 127.65, 126.84, 126.75, 126.73, 126.36, 126.02, 123.31, 118.25, 117.68, 31.00.

The method of fibrils formation

Insulin amyloid fibrils were prepared according to the protocols described elsewhere.[1] A stock solution containing Insulin (5 mg • mL^{-1}) and HCl (0.01 M, pH = 2) were prepared. A HCl (0.01 M, pH = 2) solution containing Insulin (5 mg • mL^{-1}) and NEB (350 μM) were prepared. The solutions were incubated in an orbital thermomixer with constant agitation at 600 rpm at 60 ℃ for 24 h.

Optical Measurement

In the study of using NEB and ThT as an ex situ probe, an aliquot of the insulin solution taken out from the incubation mixture at a defined time was diluted with Tris-HCl buffer, followed by the addition of the probe. The final concentrations of insulin, NEB and ThT were 5 μM, 2 μM and 2 μM, respectively.

In the study of using NEB and ThT as an in situ probe， NEB and ThT was added to insulin solution prior to incubate at 60 °C with constant agitation at 600 rpm. Then an aliquot of the insulin solution with the probe taken out from the incubation mixture at a defined time was diluted with Tris-HCl buffer. The final concentrations of insulin, NEB and ThT were 5 μM, 2 μM and 2 μM, respectively.

Transmission electron microscopy (TEM) experiment.

In order to confirm the existence of fibrils, the solution of insulin was incubate 24 h, which examined by TEM. The solution was diluted 20 times with 10 mM Tris – HCl buffer and a drop placed on a Formvar-coated grid. The resulting grid, after drying, was studied using a Hitachi H-7650 transmission electron microscopy, operating with an accelerating voltage of 80 kV.

The method of measuring the dissociation constant

The dissociation constants (K_d) of NEB and ThT were derived by using a fixed concentration of the mature insulin fibrils with varying dye concentrations and fitted using a one site binding equation. The resulting K_d for NEB and ThT are 3.36 μM and 6.68 μM, respectively.

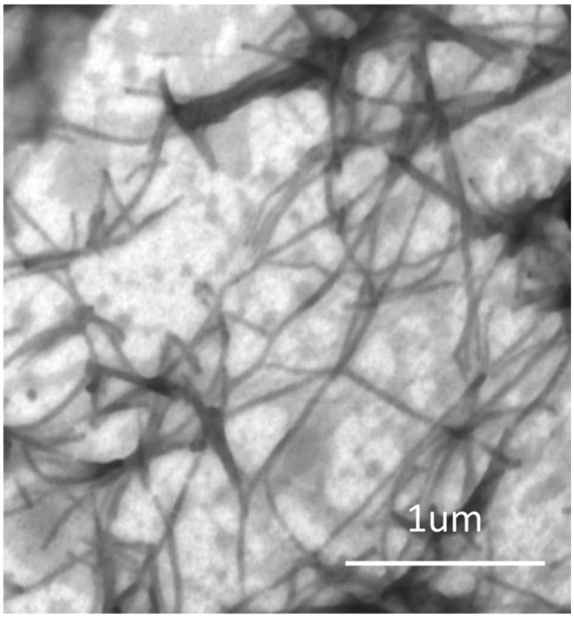

Figure S1 TEM image of fibrils of insulin. The fibrils have a length of several micrometres with an average width of 7-10 nm.

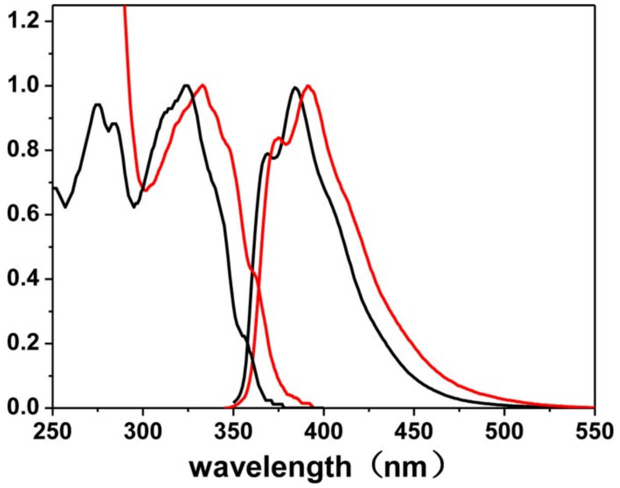

Figure S2 Normalized absorption and emission spectra of NEB alone in water solution (black) and NEB bound to insulin fibrils (red). The absorption spectrum has a 9 nm red-shift from 324 nm to the 333 nm and the fluorescence spectrum exhibits a 7 nm red-shift from 384 nm to the 391 nm.

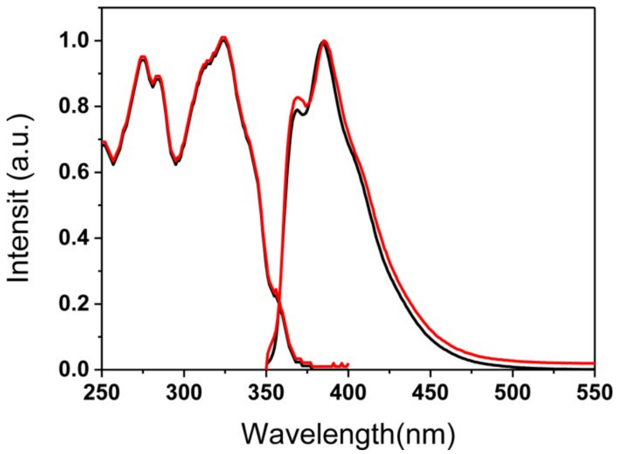

Figure S3 Normalized absorption and emission spectra of NEB alone in water solution (black) and NEB alone in native insulin solution (red). We compared the absorption and fluorescence spectra of NEB containing insulin before fibrillation with that of NEB in water solution and no difference is found. This results indicate that NEB is binding specifically to insulin fibrils and not to native protein.

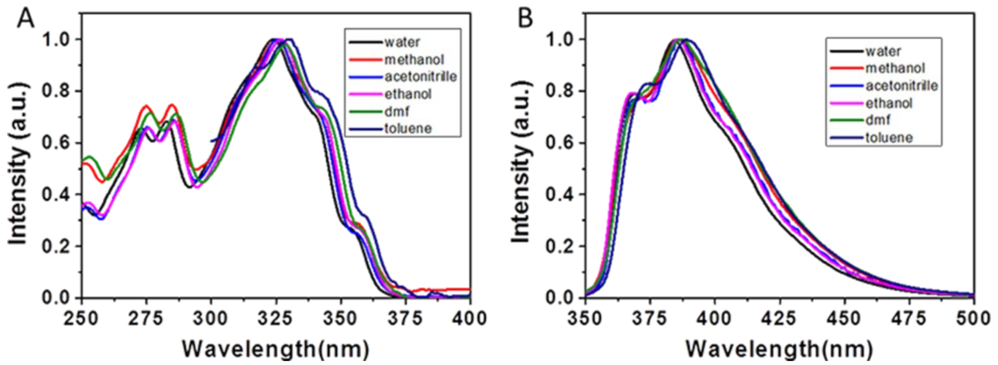

Figure S4 The emission Normalized absorbance and emission spectra of NEB in different solution polarity.

Table 2. Photophysical datas for NEB in different solvents

solvents	water	methanol	acetonitrile	ethanol	dmf	toluene
**λabs**	324	325	326	327	330	331
**λem**	384	386	386	386	386	389
Δf	0.320	0.309	0.306	0.290	0.275	0.236

Δf =(ε-1)/(2 ε +1)-(n2-1)/(2n2+1) is the solvent orientational polarazability.

ε is the dielectric constant and n is the refraction index.

- The maximum absorption peak shifts from 324 nm (water) to 331nm (toluene), showing a red-shit of 5 nm. Correspondingly, the fluorescence emission maximum shifts from 384 to 389 nm with reducing polarity of the solvent.

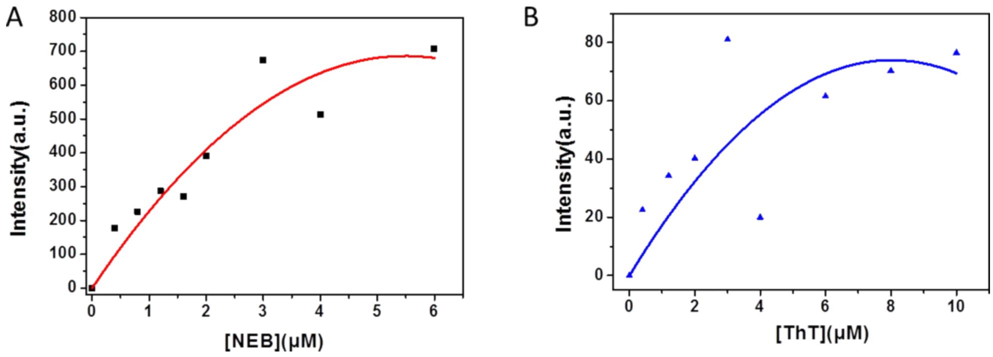

Figure S5 The saturation binding of NEB to fibrillar form of insulin (0.08 μM). The dissociation constants (K_d) of NEB and ThT fitted using a one site binding equation. The resulting K_d for NEB and ThT are 3.36 μM and 6.68 μM, respectively.

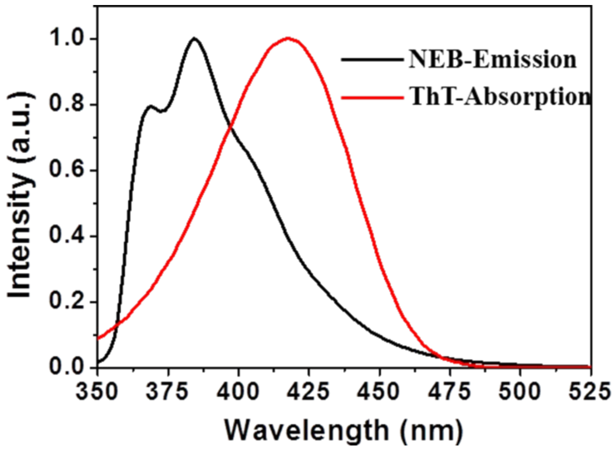

Figure S6 Normalized absorption of ThT (red) and emission of NEB (black) spectra of NEB alone in solution. The emission of NEB display bluish emissive, with a broad spectrum covering 350–500 nm that overlaps absorption spectrum of ThT.

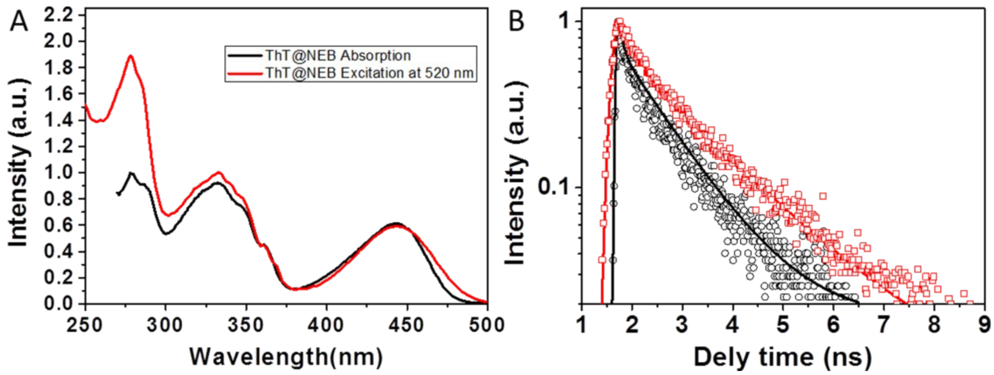

Figure S7 (A). The normalized absorption spectra of the sample NEB @ ThT and the excitation spectra of the sample NEB @ ThT at λ_{em} = 520 nm (red). (B). Fluorescence decay profiles of NEB in mature fibrils solution (red line), and mature fibrils solution added ThT(black line).

- The absorption spectra of the sample NEB @ ThT exhibits the absorption peaks with a strong peak at 325 nm ascribed to the NEB and a relatively weaker peak at 410 nm from the ThT. Fluorescence excitation spectrum monitored at the ThT emission (520 nm) agree well with the absorption spectra. energy transfer may takes place from excited NEB to ThT.
- The average lifetime of NEB bound to insulin fibrils alone decays 1.42 ns. The fluorescence decay of NEB in the NEB @ ThT bound to mature insulin fibrils becomes faster, giving an average lifetime of 0.51 ns. All of these proved that efficient energy transfer takes place from excited NEB to ThT.

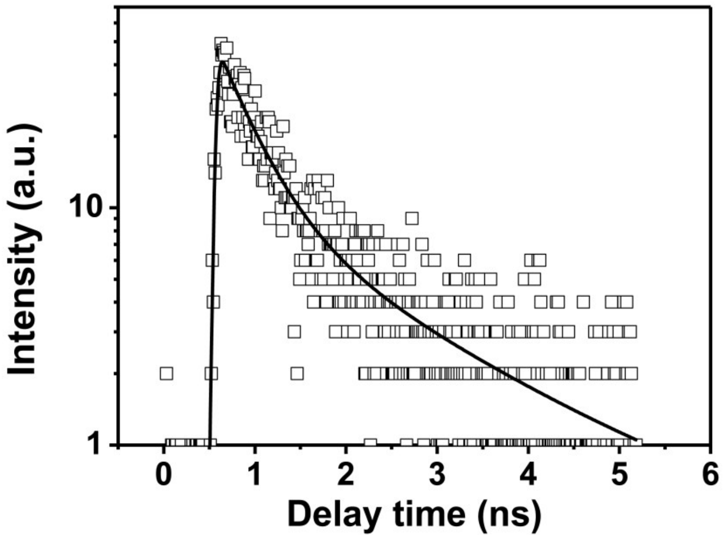

Figure S8. Fluorescence decay profiles of NEB during the incubation process (incubation time 6 h).

- After incubating for 6 h, the time resolved fluorescence decays of the NEB bound to insulin fibrils was also represented by a double-exponential fit with a lifetime of 0.41 ns (A1 = 0.78) and a slower component 1.82 ns (A2 = 0.22). The average excited-state lifetime is 0.72 ns, which is slower than the NEB in water alone （0.50 ns），and faster than NEB binding to the mature fibrils. Comparing to the NEB in water alone, the value of k_r increases to 0.42 ns^{-1} with the value of k_{nr} decreases to 1.0 ns^{-1}. These results verified that the fluorescence enhancement by the restriction of the NEB's internal rotation around the C-C bond when bound to the insulin fibrils.

1 C. C. Kitts, T. Beke-Somfai, B. Nordén, biochem., 2011, 50, 3451-3461.
